# OMIT: Dynamic, Semi-Automated Ontology Development for the microRNA Domain

**DOI:** 10.1371/journal.pone.0100855

**Published:** 2014-07-15

**Authors:** Jingshan Huang, Jiangbo Dang, Glen M. Borchert, Karen Eilbeck, He Zhang, Min Xiong, Weijian Jiang, Hao Wu, Judith A. Blake, Darren A. Natale, Ming Tan

**Affiliations:** 1 School of Computing, University of South Alabama, Mobile, Alabama, United States of America; 2 Corporate Technology, Siemens Corporation, Princeton, New Jersey, United States of America; 3 Department of Biology, University of South Alabama, Mobile, Alabama, United States of America; 4 School of Medicine, University of Utah, Salt Lake City, Utah, United States of America; 5 The Jackson Laboratory, Bar Harbor, Maine, United States of America; 6 Georgetown University Medical Center, Washington, DC, United States of America; 7 Mitchell Cancer Institute, University of South Alabama, Mobile, Alabama, United States of America; King's College, London, United Kingdom

## Abstract

As a special class of short non-coding RNAs, microRNAs (a.k.a. miRNAs or miRs) have been reported to perform important roles in various biological processes by regulating respective target genes. However, significant barriers exist during biologists' conventional miR knowledge discovery. Emerging semantic technologies, which are based upon domain ontologies, can render critical assistance to this problem. Our previous research has investigated the construction of a miR ontology, named Ontology for MIcroRNA Target Prediction (OMIT), the very first of its kind that formally encodes miR domain knowledge. Although it is unavoidable to have a manual component contributed by domain experts when building ontologies, many challenges have been identified for a completely manual development process. The most significant issue is that a manual development process is very labor-intensive and thus extremely expensive. Therefore, we propose in this paper an innovative ontology development methodology. Our contributions can be summarized as: (i) We have continued the development and critical improvement of OMIT, solidly based on our previous research outcomes. (ii) We have explored effective and efficient algorithms with which the ontology development can be seamlessly combined with machine intelligence and be accomplished in a semi-automated manner, thus significantly reducing large amounts of human efforts. A set of experiments have been conducted to thoroughly evaluate our proposed methodology.

## Introduction

Prior research, [1] [2] for example, has demonstrated that microRNAs (a.k.a. miRNAs or miRs), a special class of short non-coding RNAs, perform important roles in various biological processes by regulating their respective target genes. To completely understand and fully delineate miR functions, an effective bio-curation is indispensable, and the curation in turn relies on effective knowledge discovery and unification from various data sources. Conventionally, biologists need to query PubMed [3] and TarBase [4] for biologically validated miR targets and various prediction databases/websites (TargetScan [5], miRDB [6], and miRGator [7] for example) for computationally putative targets. More often than not, biologists also need to extract additional information for each and every miR target, either validated or putative, with regard to its protein functions, affiliated signaling pathways, and so forth. Therefore, biologists are required to explore large amounts of data sources and identify possible links among these data sources.

### Significant Barriers in Conventional miR Knowledge Discovery

The aforementioned data sources were developed by different research groups around the world. Thus, involved data are inherently heterogeneous in their semantics (intended meaning). If there are no common standards to represent disparate sources it will be extremely challenging to connect heterogeneous data sources with each other. Unfortunately, naming conventions for miRs are in their infancy and not uniformly adopted. Therefore, miR nomenclature has not yet been effectively standardized and the entity naming remains largely attached to their authors' favorite choices. The complex terminologies, along with their heterogeneous semantics, have led to significant barriers during conventional miR knowledge discovery and unification, which is time-consuming, labor-intensive, error-prone, and subject to biologists' limited prior knowledge.

### The Need to Have a miR Domain Ontology

Emerging semantic technologies have been widely applied in biomedical and biological area. Inspired by previously successful examples, including Gene Ontology (GO) [8], [9], Sequence Ontology (SO) [10], [11], and PRotein Ontology (PRO) [12] among others, it is reasonable to assume that semantic technologies can render critical assistance in miR knowledge discovery as well. Since semantic technologies are based upon domain ontologies, in our previous research [13], [14] we investigated the construction of a miR ontology, named Ontology for MIcroRNA Target Prediction (OMIT), the very first of its kind that formally encodes miR domain knowledge. OMIT was meant to fill the gap of lacking specific miR domain ontologies. Consequently, a standardized miR metadata model and common data elements were provided to enable data connections among heterogeneous sources, leading to more effective miR data integration and knowledge discovery [15], [16].

### The Need to Develop the Ontology in a (Semi)Automated Manner

Despite the fact that it is essential to have a manual component contributed by domain experts when building ontologies, prior research [17]–[19] has demonstrated that a “purely” manual ontology development has many drawbacks, including but not limited to, being significantly labor-intensive and extremely expensive in all aspects. Therefore, we propose in this paper a semi-automated methodology to construct domain ontologies. Our method makes use of machine intelligence, considers miR domain-dependent and domain-independent properties/relationships, is scalable, and will significantly reduce human efforts.

The rest of this paper is organized as follows. Section “Related Work” provides a summarization of state-of-the-art research in computational identification of miR target genes, biomedical and biological ontologies, and automated ontology development, respectively; Section “Materials and Methods” describes in detail the proposed methodology, including the development of a backbone ontology, the ontology/schema alignment algorithm, and the augmentation of the backbone ontology; Section “Experimental Results and Analysis” reports experimental results along with in-depth discussions; Section “Materials in Greater Details” contains greater details of related work and our methods for readers' reference; and finally, Section “Conclusions” concludes with future research directions.

## Related Work

In this section, we briefly discuss the current status of three areas that are related to this paper: (i) computational identification of miR target genes, (ii) biomedical and biological ontologies, and (iii) automated ontology development.

### Computational Identification of miR Target Genes

The principal goal of various miR target prediction approaches [20]–[33] is to reduce the prohibitively large numbers of predicted targets. (a) The degree of target site conservation and (b) a target's involvement in a pathway where other targets are also predicted are just two examples of legitimate considerations for refining miR target predictions. Additionally, binding of miR:mRNA pairs is affected by spatial and temporal co-expression of the miR:mRNA pair, as well as the target site availability. The formation of a stable duplex at the target site also plays a role in target site determination. As the determination of the co-expression of miR:mRNA pairs is becoming a reality through next generation sequencing of mRNA-enriched libraries and small RNA libraries from the same cells, prediction tools can now be cross-referenced with expression data. To the best of our knowledge, there are more than 20 distinct miR target prediction tools. A list of currently available tools is provided in Table 1, including detailed information for each tool such as the prediction strategy and available access method.

**Table 1:** A List of Current miR Target Prediction Tools.

Prediction Tool Name	Prediction Strategy	Access	Official Website
deepBase	A database for annotating and discovering small and long ncRNAs (microRNAs, siRNAs, piRNAs…) from high-throughput deep sequencing data.	Both	http://deepbase.sysu.edu.cn/
DIANA-microT-CDS	Thermodynamic modeling.	Both	http://diana.cslab.ece.ntua.gr/
DIANA-mirGen 2.0	A database of microRNA genomic information and regulation.	Both	http://diana.cslab.ece.ntua.gr/mirgen/
GenMiR++	Paired expression profiles of microRNAs and mRNAs; as well as Baynesian inference.	Both	http://www.psi.toronto.edu/genmir
mimiRNA	Expression correlation.	Both	http://mimirna.centenary.org.au
mirBridge	Complementary and target site conservation.	Download	http://mirbridge.org/
miRanda	Complementary and target site conservation.	Both	http://www.microrna.org
miRBase	A searchable database of published miRNA sequences and annotation.	Both	http://www.mirbase.org/
miRDB	Microarray corrleation training; as well as Support Vector Machine.	Both	http://mirdb.org
miRecords	Validated targets and algorithm integration.	Both	http://mirecords.biolead.org/doc.php
miRGator	Expression correlation and algorithm integration.	Online Search	http://mirgator.kobic.re.kr/
miRGen	Positional relationships target prediction integration.	Both	http://www.diana.pcbi.upenn.edu/miRGen.html
miRNA-Target Gene Prediction at EMBL	Complementary and target site conservation.	Online Search	http://www.russell.embl.de/miRNAs
miRNAMap	Genomic maps of microRNA genes and their target genes in mammalian genomes.	Both	http://mirnamap.mbc.nctu.edu.tw/
MicroInspector	Algorithm integration.	Online Search	http://bioinfo1.uni-plovdiv.bg/cgi-bin/microinspector/
MiTarget	Positional relationships thermodynamic modeling; as well as Support Vector Machine.	Online Search	http://cbit.snu.ac.kr/miTarget
PicTar	Target site conservation and thermodynamic modeling.	Both	http://pictar.mdc-berlin.de/
PITA	Incorporating the role of target site accessibility, as determined by base-pairing interactions within the mRNA, in microRNA target recognition.	Both	http://genie.weizmann.ac.il/
PMRD	PMRD: Plant microRNA database.	Both	http://bioinformatics.cau.edu.cn/PMRD/
RepTar	Searching for repeating 3′ UTR target sites.	both	http://reptar.ekmd.huji.ac.il/
RNA22	Identifying patterns in cDNAs and matching to miRs.	Online search	http://cbcsrv.watson.ibm.com/rna22.html
RNAhybrid	Thermodynamics & statistical model.	Both	http://bibiserv.techfak.uni-bielefeld.de/rnahybrid
starBase	Argonaute CLIP-Seq and degradome sequencing data.	Both	http://starbase.sysu.edu.cn/
TarBase	Experimentally validated targets.	Both	http://diana.cslab.ece.ntua.gr/tarbase/
TargetScan	Seed complementary and target site conservation.	Both	http://www.targetscan.org
ViTa	Complementary of host microRNAs to viruses.	Both	http://vita.mbc.nctu.edu.tw/

### Existing Biomedical and Biological Ontologies

Ontologies have been used for a long time to describe entities for some domain in a formal manner, as well as relationships among these entities. In general, an ontology consists of a collection of well-defined concepts (a.k.a. terms or classes), properties of these concepts, relationships among concepts, and some constraints on concepts, properties, and relationships. The ontology structure (a.k.a. schema) usually well reflects a representation or encoding of intended domain knowledge meant by respective ontology developers, thus serving as a guide for better organizing data and turning data into knowledge. Ontologies and semantic technologies have been widely utilized in biomedical and biological research. Biomedical and biological ontologies are often referred to as bio-ontologies, and they have become increasingly popular nowadays. Existing bio-ontologies [8]–[12], [34]–[36] not only have demonstrated the necessity and importance to apply semantic technologies in biomedical and biological area, but also have provided us with critical resources during the miR ontology development (detailed in Section “Materials and Methods”).

### (Semi)Automated Ontology Development

During the ontology development, on one hand, a manual component from domain experts is considered unavoidable to effectively encode precise semantics; on the other hand, many drawbacks have been identified if we adopt a completely manual ontology construction process [17], [18]. The most significant challenge is that a manual development process is extremely labor-intensive and highly expensive, referred to as the knowledge discovery bottleneck [19]. As a result, (semi)automated ontology development has attracted a large amount of research. Existing algorithms can be divided into three categories: translation-based [37]–[40], mining-based [41]–[47], and external knowledge-based [48]–[52]. Despite its importance, much more progress is still needed in (semi)automated ontology development. In particular, while *is_a* is the most common and critical ontological relationship, the importance of other relationships, especially those domain-dependent relationships, has been historically underestimated in many state-of-the-art algorithms. Additionally, existing algorithms, if based on machine-learning technologies, tend to focus on ontological instances. Unfortunately, many real-world ontologies have few or no instances at all. For example, GO, the most successful bio-ontology, does not have any instances [53].

## Materials and Methods

### Three-Step Semi-Automated Ontology Development Process

**Figure 1:**
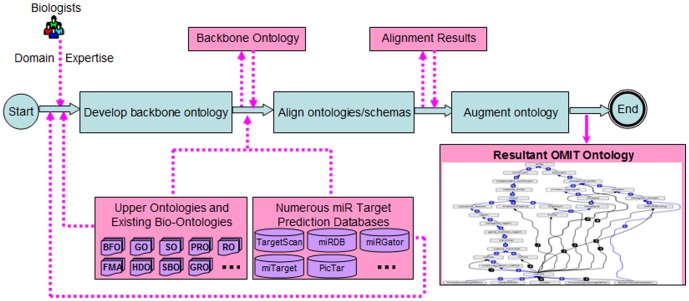
Three steps in the proposed semi-automated ontology development: (i) develop a backbone ontology; (ii) align the backbone ontology with other ontologies/schemas; and (iii) augment the backbone ontology.

As demonstrated in Figure 1, the semi-automated ontology development consists of three steps.

- **Step 1.** Construct an initial “backbone” miR ontology using a knowledge-driven approach, which is iterative and combines both top-down and bottom-up processes. Domain expertise, popular upper ontologies, existing bio-ontologies, and current miR target prediction databases will be made use of, and widely-accepted development principles and procedures will be adopted.
- **Step 2.** Design an algorithm to align the backbone ontology with existing bio-ontologies and numerous miR target prediction databases. The algorithm is based on artificial neural networks (ANNs) and agglomerative clustering, and will learn from the schema level (either ontology structures or database schemas) instead of from the instance level. Additionally, many properties and relationships, those miR domain-dependent ones in particular, will be taken into account besides the *is_a* relationship. The alignment results are equivalent concept pairs among different ontologies/schemas.
- **Step 3.** According to the obtained equivalent concept pairs, it is straightforward to append additional entities (i.e., concepts along with their properties, relationships, possible instances, and direct/indirect descendant concepts) from one ontology/schema into another one. The initial backbone ontology will thus be augmented by ontological entities from other ontologies/schemas.

### Backbone Ontology Development

Following the literature in ontology development [54]–[57], we have adopted an iterative procedure, combining both top-down and bottom-up processes, to construct the initial backbone ontology. The top-down process starts with the definition of most general concepts followed by specialization of these concepts. Popular upper ontologies and existing bio-ontologies have been utilized. The bottom-up process starts with the definition of most specific concepts followed by grouping of these concepts into more general concepts. Schemas and instances from current miR target prediction databases have been consulted. Both top-down and bottom-up processes have relied on miR domain expertise offered by two experimental biologists (both are co-authors of this paper).

#### Data Sources

Three types of data sources have been made use of.

- Popular upper ontologies. In particular, we have used Basic Formal Ontology (BFO) [58]–[60] to describe general concepts that are the same across all knowledge domains. BFO is the only upper ontology that is currently adopted in Open Biomedical Ontologies (OBO) Foundry/Library bio-ontologies. Adopting BFO concepts will help agree on structures and relationships that can be reused across multiple ontologies, and thus avoid the necessity to repeatedly consider general concepts in ontology construction efforts. Note that there is a tradeoff in using BFO concepts since BFO is mainly meant for use by ontology engineers. Fortunately, BFO concepts can be easily stored into a separate file using OWL's axiom-level modularity [61] and thus hidden to non-expert users. Based on this analysis, we have decided to include the BFO structure in OMIT.
- Existing bio-ontologies, such as GO, SO, PRO, OBO Relation Ontology (RO) [36], Foundational Model of Anatomy (FMA) [62], Human Disease Ontology (HDO) [63], System Biology Ontology (SBO) [64], and Gene Regulation Ontology (GRO) [65]. The purpose is not only to reduce possible redundant efforts in the ontology development, but also to achieve a better orthogonality with existing bio-ontologies. In particular, we have placed special emphasis on well-established bio-ontologies under the OBO Foundry/Library, a resource for ontologies shared across different biological and biomedical domains.
- Current miR target prediction databases. Out of more than 20 distinct miR target prediction tools, we have prioritized and selected six databases, i.e., DIANA-microT [66], [67], miRanda [20]–[24], miRDB [68], [69], miRGen [70], TarBase [4], and TargetScan [26]–[28], based on numerous considerations: the quality of database instances; previous research collaboration; a convenient, up-to-date data download mechanism; and the popularity of the database.

#### Ontology Development Principles and Procedure

We have observed seven practices proposed by OBO Foundry Initiative [71]. The ontology should be freely available, expressed in a standard language, documented for successive versions, orthogonal to existing ontologies, including natural language specifications, developed collaboratively, and used by multiple researchers. The ontology development procedure consists of three main steps as follows.

1. Computer scientists (i.e., ontology engineers) work together with domain experts (i.e., experimental molecular biologists) to specify the range of concepts to be included in the ontology.
2. Definitions of these identified concepts are formalized using Description Logic and documented.
3. Concepts along with their properties and relationships are implemented in computer languages.

**Figure 2:**
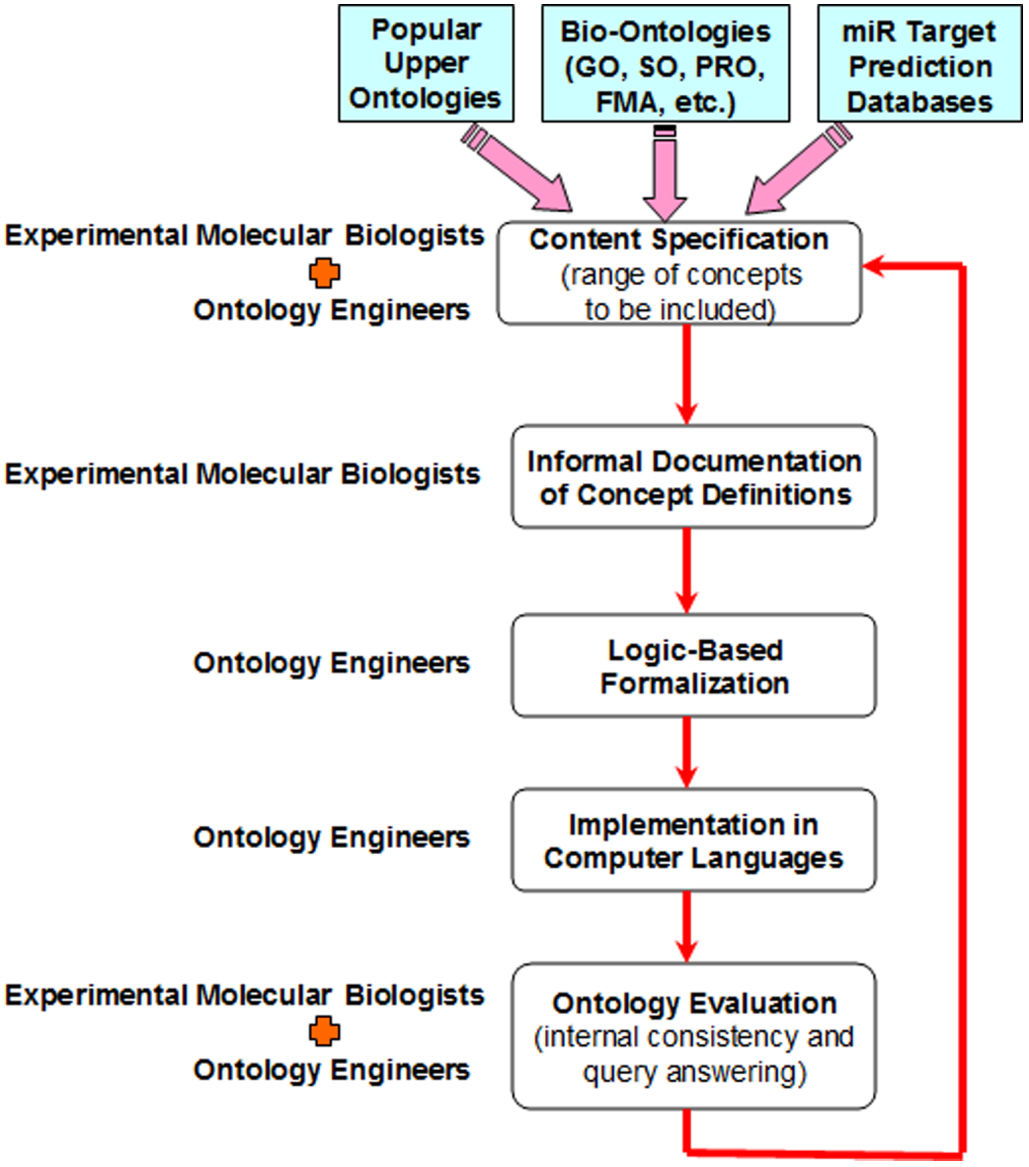
The development of a backbone ontology.

A flowchart is exhibited in Figure 2. The development procedure is in fact an iterative one in that we have solicited feedback, verification, and evaluation from domain experts and then incorporated their opinions and suggestions on a regular basis and in a structured manner.

#### Ontology Format/Language and Development Tool

There are different formats and languages for describing ontologies, all of which are popular and based on different logics: Web Ontology Language (OWL), OBO, Knowledge Interchange Format (KIF), and Open Knowledge Base Connectivity (OKBC). We have chosen both the OWL and OBO formats, and our choice was based upon the following observations. OWL was recommended by the World Wide Web Consortium (W3C) and was designed for use by applications that need to process the content of information instead of just presenting information to humans. As a result, OWL facilitates greater machine interpretability of Web contents. OBO is widely used in the bio-ontology community, and many well-developed bio-ontologies, especially those in OBO Foundry/Library, have adopted the OBO format. As for the development tool, we have chosen Protégé [72], [73] and OBO-Edit [74], [75] over other available tools such as CmapTools [76] and OntoEdit [77]. In addition, we have followed a set of well-established naming conventions for various ontological entities. Whenever possible, we have supplied human-readable definitions for concepts, properties, and relationships. These definitions concisely and clearly state respective semantics. Greater details of ontological naming conventions and human-readable definitions can be found in Section “Materials in Greater Details.”

### Ontology/Schema Alignment Algorithm

The proposed alignment algorithm is based on machine-learning technologies. We aim to tackle two challenges in state-of-the-art algorithms: (i) lacking sufficient instance data to learn from and (ii) underestimated importance of properties and relationships other than the *is_a* relationship. Given a pair of ontologies/schemas, it is reasonable to assume that contributions from different semantic aspects (i.e., concept names, concept properties, and various relationships) would hold across and therefore be independent of specific concepts. In fact, these contributions are characteristics of specific ontologies/schemas (viewed as a whole) and thus become the foundation for corresponding semantic weights. In other words, during the ontology/schema alignment, semantic weights are determined by respective ontologies/schemas rather than by individual concepts. It is thus possible to *learn these weights for all concepts by training examples from a subset of concepts*. This assumption will be verified later in Section “Experimental Results and Analysis.”

#### Calculating Semantic Similarity

Between a pair of concepts, 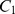 and 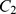, a total of four semantic similarity measures have been designed.

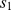
 represents the similarity on the concept name. First, hyphens and underscores are removed and nouns are transformed from their plural forms to single forms. Upon completion of such pre-processing, if two names have an exact string matching or are synonyms of each other in WordNet [78] then 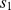 has a value of 1. Otherwise, 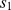 is calculated as 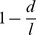, where 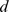 stands for the edit distance between two strings, and 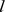 stands for the length of the longer string.

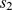
 represents the similarity on the concept property list, calculated by the percentage of matched properties between 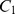 and 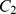. The principle of “stable marriage” is adopted during the calculation: once a property from the first concept is matched with another property from the second concept, neither property will be considered anymore. Additionally, many domain-dependent properties specifically designed for the miR field are considered, such as *cellLines*, *chromosomeLocation*, *miRNATargetSequence*, *miRNATargetGeneSymbol*, and *miRNATargetCompleteName*.

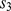
 represents the similarity on the *is_a* relationship. First, two ancestor lists, *ancestor* concepts of 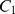 and *ancestor* concepts of 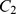, are calculated. Pairwise matching will then be performed among concepts from these two lists, and likewise, using the principle of stable marriage (once a concept from the first list is matched with another concept from the second list, neither concept will be considered anymore). After pairwise similarities between two ancestor lists are obtained, the average value of these similarities is calculated as 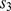 between 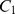 and 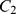.

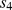
 represents the similarity on *hasBinding*, a domain-dependent relationship specifically designed for the miR field, calculated by the percentage of matched concepts between *hasBinding* concepts of 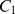 and *hasBinding* concepts of 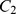. Similarly, the principle of stable marriage is adopted.

#### Weight Learning and Agglomerative Clustering

After four similarity values are obtained, an overall similarity, 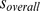, between two concepts is calculated as the weighted sum of 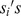, i.e., 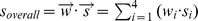. Next, a matrix of the overall similarity (short for “similarity matrix” in the rest of this paper) between pairwise concepts is created. Initially, 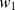 through 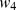 are randomly set to some values. We then utilize an ANN to learn optimal weights, that is, to find the weight vector (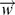) that best fits training examples. A formal defintion of the learning problem, the search strategy within the hypothesis space, and the pseudocode for the weight-learning algorithm are provided in Section “Materials in Greater Details.”

Once the optimal 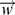 is obtained from the ANN learning, the similarity matrix is recalculated with updated weights. An agglomerative clustering algorithm is then utilized to generate equivalent concept pairs. Initially, each concept is regarded as a *singleton* cluster, and clusters of two equivalent concepts can be merged with each other and form a new cluster. New clusters continue to be generated until the maximum similarity between any two clusters is below a predefined threshold. Finally, newly generated clusters are output as the set of equivalent concept pairs. The pseudocode is provided in Section “Materials in Greater Details.”

Due to the low time complexity of both weight-learning and clustering algorithms (detailed analysis can be found in Section “Materials in Greater Details”), the proposed semi-automated ontology development has better efficiency and scalability than a completely manual development. Additionally, this conclusion will be further verified by our experimental results in Section “Experimental Results and Analysis.”

### A Cycle of Iterative, Dynamic Improvement of OMIT

The proposed semi-automated ontology development is essentially an iterative and dynamically improved process. After the backbone ontology was constructed and aligned with other ontologies/schemas, the first version of OMIT was generated by augmenting ontological information from other data sources. We then solicited verification and evaluation from domain experts; their opinions and suggestions were in turn incorporated into the next version of OMIT. Such a feedback and enhancement mechanism has been performed on a regular basis and in a structured manner. Consequently, the ontology has been iteratively and dynamically improved over time. We have adopted revision-control procedures to document the process for future reference. Microsoft Visual SourceSafe (MVSS) [79] has been selected over other available tools such as Concurrent Versions System (CVS) [80] and Revision Control System (RCS) [81].

## Experimental Results and Analysis

### Experimental Environment

All experiments were conducted on personal computers with the following configuration: Intel(R) Core(TM) i7-3632 QM CPU @ 2.20 GHz 2.20 GHz; 8.00 GB memory; and Windows 7 64-bit Operating System.

### Backbone Ontology

The backbone ontology contains a total of 53 concepts, 12 properties, and 17 relationships (besides *is_a*).

- Example concepts include *miRNA*, *gene_expression*, *Tumor*, *Organ*, *object*, *material_entity*, *independent_continuant*, *continuant*, and *entity*. Greater details of these concepts are exhibited in Table 2.
- Example properties include *cellLines*, *chromosomeLocation*, *directSupport*, *experimentSummary*, *miRNACompleteName*, *miRNASequenceLocation*, *targetGeneSymbol*, *targetPrimaryPeptideSequence*, and *targetTermAssociations*. These properties were all specifically designed for the miR field.
- Example relationships include *is_a*, *has_part*, *part_of*, *affectsTumor*, *hasBinding*, *hasPrediction*, *hasTarget*, *hasValidation*, *involvedInEvent*, and *regulateEvent*. Greater details are exhibited in Table 3. Most relationships listed here, except for the first three, were specifically designed for the miR field.

**Table 2:** Sample Concepts in the Backbone Ontology.

Concept Name	Created by ourselves?	Imported from	Properties Extended?	Relationships Extended?
*miRNA*	No	SO	Yes	Yes
*chromoplast_gene*	No	SO	No	No
*gene_expression*	No	GO	No	Yes
*biological_process*	No	GO	No	Yes
*protein*	No	PRO	Yes	Yes
*amino_acid_chain*	No	PRO	No	Yes
*Tumor*	No	HDO	Yes	Yes
*Organ*	No	FMA	Yes	Yes
*object*	No	BFO	No	Yes
*material_entity*	No	BFO	No	Yes
*independent_continuant*	No	BFO	No	Yes
*continuant*	No	BFO	No	Yes
*entity*	No	BFO	No	Yes
*MiRNABinding*	Yes	N/A	N/A	N/A
*ExperimentalValidation*	Yes	N/A	N/A	N/A
*PharmaceuticalTreatment*	Yes	N/A	N/A	N/A
*AdvantageousRegulation*	Yes	N/A	N/A	N/A
*OncoGeneratingMiRNA*	Yes	N/A	N/A	N/A
*TumorSuppressingMiRNA*	Yes	N/A	N/A	N/A
*OtherMiRNA*	Yes	N/A	N/A	N/A

**Table 3:** Sample Relationships in the Backbone Ontology.

Relationship Name	Simple Definition or Usage	miR Specific?
*is_a*	imported from OBO Relation Ontology (RO)	No
*has_part*	imported from OBO Relation Ontology (RO)	No
*part_of*	imported from OBO Relation Ontology (RO)	No
*affectsTumor*	miRs affect numerous tumors, including cancers	Yes
*hasBinding*	each miR has some mRNA binding sites	Yes
*hasPrediction*	each miR has one or more computationally predicted target genes	Yes
*hasTarget*	each miR has one or more target genes	Yes
*hasValidation*	each miR has one or more biological validations for each of its target genes	Yes
*involvedInEvent*	miRs are involved in some pathological events	Yes
*regulateEvent*	miRs can down-regulate or up-regulate some pathological events	Yes

### Ontology/Schema Alignment Results

**Figure 3:**
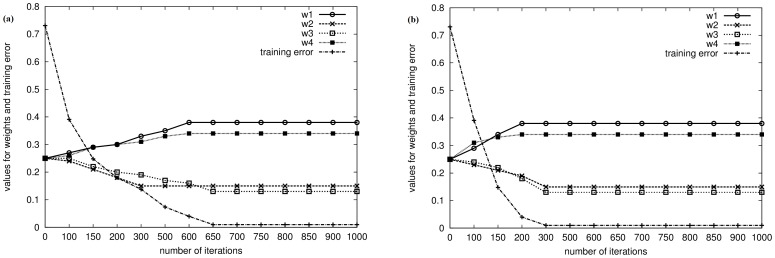
Weight convergence experimental results when aligning TarBase with the backbone ontology, where 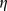 was set to 0.1 in (a) and 0.3 in (b), respectively.

We chose three ontologies/schemas to thoroughly evaluate the alignment algorithm: System Biology Ontology (SBO) [64], Gene Regulation Ontology (GRO) [65], and TarBase [4], all of which are either real-world bio-ontologies or frequently utilized biomedical/biological databases that contain miR data. The characteristics of these test ontologies/schemas are summarized in Table 4. The alignment algorithm was performed between pairwise ontologies/schemas among SBO, GRO, TarBase, and the backbone ontology, resulting in a total of six sets of experiments. Experimental results are reported in Figure 3 and Table 5.

**Table 4:** Characteristics of Test Ontologies/Schemas.

Features	SBO	GRO	TarBase
Number of Instances	0	4	0
Number of Concepts	604	507	76
Number of Properties	0	9	19
Number of Relationships	1	24	23
(excluding *is_a*)			

**Table 5:** Pairwise Alignment Results among Four Ontologies/Schemas.

	GRO + SBO	GRO + TarBase	SBO + TarBase	GRO + Backbone	SBO + Backbone	TarBase + Backbone
Initial weights	0.25 0.25 0.25 0.25	0.25 0.25 0.25 0.25	0.25 0.25 0.25 0.25	0.25 0.25 0.25 0.25	0.25 0.25 0.25 0.25	0.25 0.25 0.25 0.25
Training examples	5	2	2	5	4	3
Learned weights	0.65 0.00 0.35 0.00	0.67 0.05 0.28 0.00	0.58 0.00 0.42 0.00	0.51 0.03 0.46 0.00	0.61 0.00 0.39 0.00	0.38 0.15 0.13 0.34
Output equivalent	51	6	7	39	27	56
concept pairs (*n*_1_)						
Correct equivalent	41	5	5	33	21	49
concept pairs (*n*_2_)						
Missed equivalent	11	2	3	8	5	9
concept pairs (*n*_3_)						
*Precision* (*p*)	80.39%	83.33%	71.43%	84.62%	77.78%	87.50%
*Recall* (*r*)	78.85%	71.43%	62.50%	80.49%	80.77%	84.48%
*F-Measure* (*f*)	79.61%	76.92%	66.67%	82.50%	79.25%	85.96%
*Overall* (*o*)	59.62%	57.14%	37.50%	65.85%	57.69%	72.41%

Note that all concept pairs, except for those in Row 3 (“Training examples”) in the above table, have been used as actual test data.

- Each of the four semantic weights, 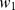, 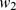, 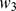, and 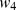, was initialized to 0.25 in all six sets.
- All weights converged to certain values in each set. This verified our hypothesis discussed earlier: different semantic weights are characteristics of specific ontologies/schemas viewed as a whole and can be learned from a subset of concepts.
- Different pairs of ontologies/schemas had different learned weights because weights reflected intended meanings encoded by original ontology/schema developers. For example, the learned 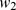 (the semantic weight of concept property similarity) for any ontology pairs involving either SBO or GRO or both was much smaller than that of other pairs; in particular, 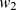 was learned as 0.00 when aligning SBO with any other ontologies/schemas. The reason is that, SBO has not defined any properties at all, and GRO has only defined nine properties (while it contains a total of 507 concepts). Another example is that, 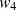 is the semantic weight of similarity on *hasBinding*; since neither SBO nor GRO defines this miR domain-dependent relationship, their corresponding 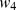 was learned as 0.00. These observations further reinforced our claim that different ontologies/schemas have different semantic weights.
- The speed of weight convergence was proportional to the learning rate 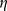, which is described in Section “Materials in Greater Details.” Figure 3 plots the weight-learning process along with the change of training error when aligning TarBase with the backbone ontology. When 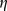 was set to 0.1 (Figure 3(a)), it took around 600 iterations during Gradient descent (the **for** loop in Line 2 in Pseudocode 1 — ANN Weight Learning) before all weights converged. As a comparison, when 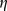 was increased to 0.3 (Figure 3(b)), the number of necessary iterations decreased to around 300.
- Four commonly adopted measures were utilized to evaluate the quality of equivalent concept pairs output from the proposed alignment algorithm:

1 Precision (*p*): the percentage of correct output equivalent concept pairs (those agreed by domain experts) over all output pairs, representing the correctness aspect of the alignment algorithm, calculated as 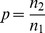 (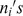 are defined in Table 5).
2 Recall (*r*): the percentage of correct output equivalent concept pairs over actually equivalent pairs, estimating the completeness aspect of the alignment algorithm, calculated as 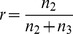 (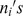 are defined in Table 5).
3 F-Measure (*f*): also referred to as *Harmonic Mean* and calculated as 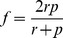, aiming to consider both Precision and Recall measures. It avoids the bias from adopting either Precision or Recall measure alone.
4 Overall (*o*): a measure calculated as 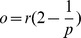, focusing on the post-alignment effort, i.e., how much human effort is needed to remove falsely aligned pairs and to add missed ones.

- Human efforts have been significantly reduced.

1 As shown in Table 5, we obtained satisfactory values in the *Overall* measure. Note that our goal was to augment the backbone ontology; therefore, the values on the last three columns should be paid closer attention to (ranging from 57.69% to 72.41%).
2 The percentage of training examples provided by domain experts over actually equivalent concept pairs (i.e., 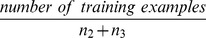, where 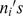 are defined in Table 5) was 9%, 28%, 25%, 12%, 15%, and 5% in each of six sets, respectively. In other words, human labor only played a small portion during the semi-automated ontology development. Likewise, the last three percentages should be paid closer attention to.
3 The number of newly added concepts was much larger than that of equivalent concept pairs (greater details are contained in the next subsection).

### Resultant OMIT Ontology

The resultant OMIT contains a total of 2,338 concepts, 39 properties, and 87 relationships (besides *is_a*). All concepts are connected into each other and form what is computationally described as a directed acyclic graph (DAG). Most concepts (around 95%) have been supplied with detailed, formal definitions and supporting documentation that can be well understood and used by non-expert users (i.e., experimental molecular biologists). Cross-referencing to related ontologies, databases, and knowledge bases is also included in OMIT, when appropriate. Compared with the backbone ontology, 2,285 concepts, 27 properties, and 70 relationships were added, all of which were augmented from real-world bio-ontologies, frequently utilized biomedical/biological databases, and miR data discussed earlier in this paper: GO, SO, PRO, OBO RO, FMA, HDO, SBO, GRO, DIANA-microT, miRanda, miRDB, miRGen, TarBase, and TargetScan. The augmentation was through the proposed methodology and verified by domain experts. Note that the number of newly added concepts was much larger than that of equivalent concept pairs output from the alignment algorithm. As discussed in Subsection “Ontology/Schema Alignment Algorithm,” direct and indirect descendant concepts were added along with identified equivalent concepts. This way, human efforts in developing domain ontologies have been significantly reduced.

**Figure 4:**
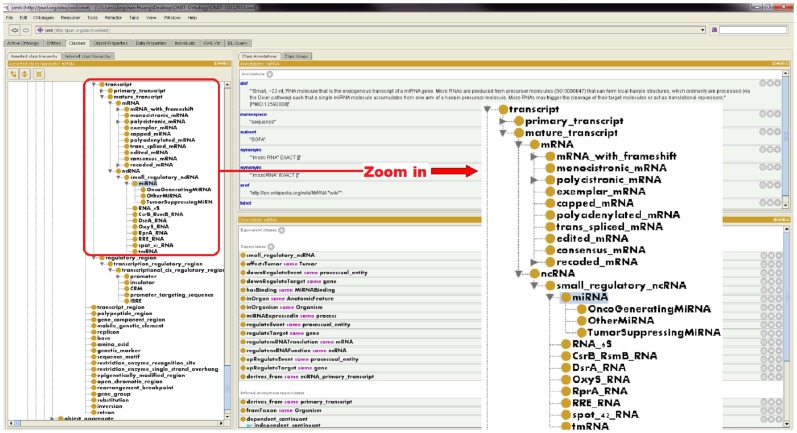
A screenshot from Protégé, demonstrating the concept *miRNA* and its parents, ancestors, descendants, and siblings in *is_a* hierarchy.

**Figure 5:**
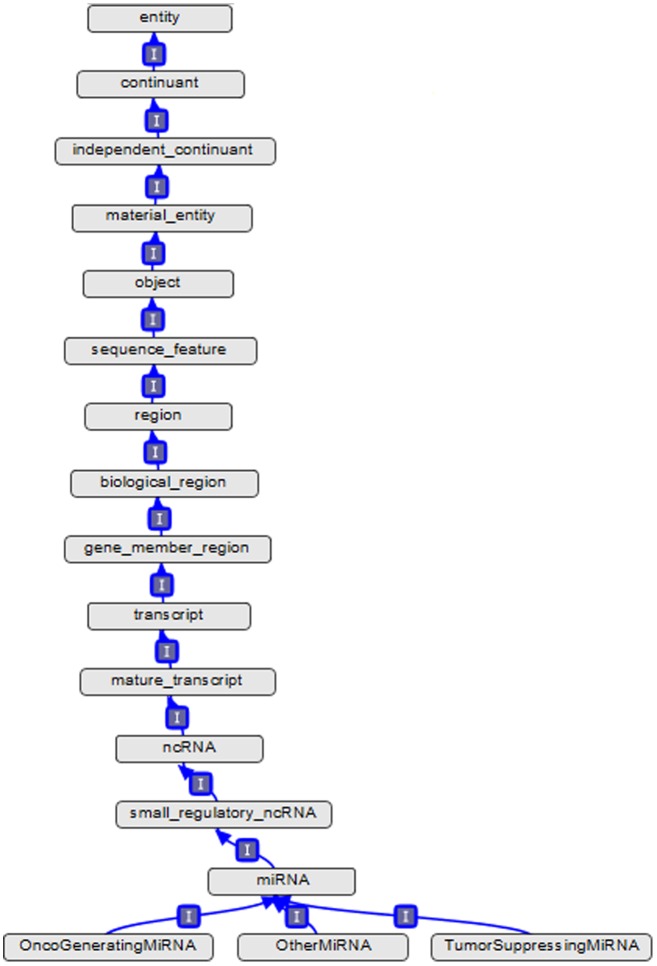
A screenshot from OBO-Edit, demonstrating more details of parents, ancestors, and direct descendants of the concept *miRNA*. All relationships exhibited in this figure are *is_a* relationships.

**Figure 6:**
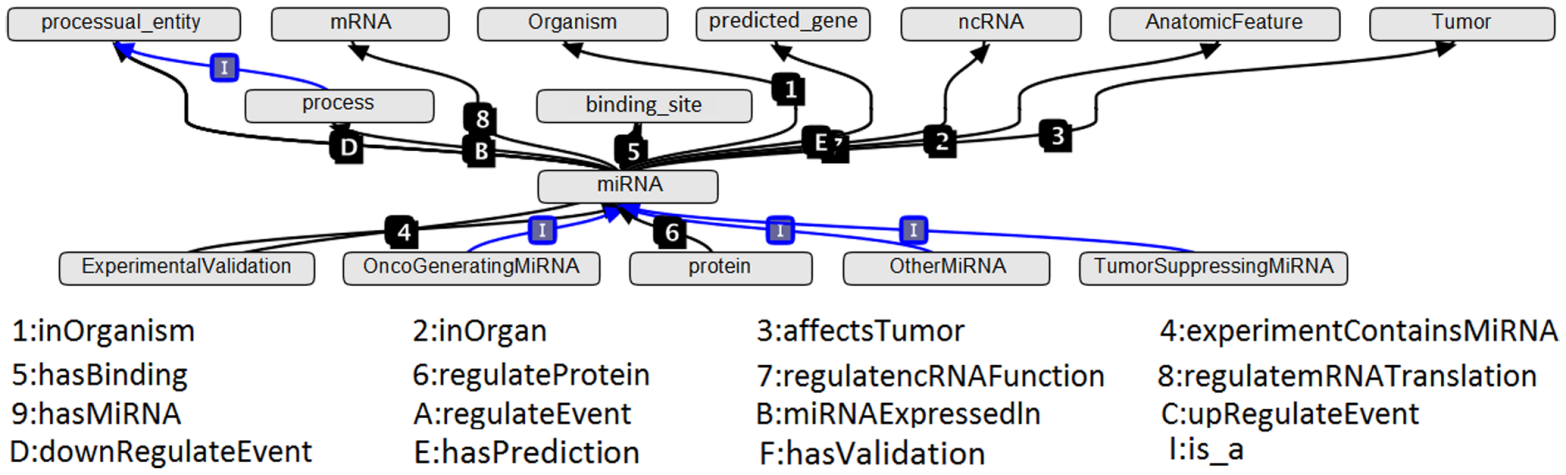
Another OBO-Edit screenshot, demonstrating a subset of relationships designed for the concept *miRNA*. Many of these relationships are miR domain-dependent ones.

Figure 4 exhibits a screenshot from Protégé, demonstrating the concept *miRNA* along with its parents, ancestors, descendants, and siblings in *is_a* hierarchy. Figure 5 exhibits a screenshot from OBO-Edit, demonstrating more details of parents, ancestors, and direct descendants of the concept *miRNA*. Figure 6 exhibits another OBO-Edit screenshot, demonstrating a subset of relationships designed for the concept *miRNA*, and many of these relationships are miR domain-dependent ones, for example:

- *affectsTumor*: each miR affects numerous tumors, including cancers
- *hasBinding*: each miR has some mRNA binding sites
- *hasPrediction*: each miR has one or more computationally predicted target genes
- *hasValidation*: each miR has various biological validations for each of its target genes

OMIT is currently included in OBO Library (http://www.obofoundry.org/cgi-bin/detail.cgi?id=omit) and NCBO BioPortal (http://bioportal.bioontology.org/ontologies/OMIT), rendered in both OWL and OBO formats. Additionally, greater technical details on our ongoing efforts, including but not limited to database files, can be found in the project website (http://omit.cis.usouthal.edu/). OMIT can be consulted any time on the Web using the aforementioned URLs. In fact, the availability of OMIT to humans and machine alike is essential in maintaining the ontology's flexibility and allowing future ontology evolution along with the increased understanding of the underlying biology.

## Materials in Greater Details

### Related Work in Computational Identification of miR Target Genes

While numerous miR target prediction algorithms have now been developed, most of these algorithms initially utilized similar sequence-based approaches to identify short complementarities between a miR and mRNA 3′ untranslated region (3′ UTR). As these complementarities are characteristically imperfect and contain mismatches, gaps, and G:U pairs, thousands of mRNAs bare alignments to any given miR identified by sequence-based approaches alone; moreover, additional steps are necessary to refine target predictions. MiRanda [20]–[24], the first publicly available miR target prediction program, refined putative target lists through calculating the thermodynamic stabilities of putative miR:target interactions by weighting C:G, A:U, and G:U pairs differently and specifically rewarding complementarities involving miR 5′ ends. Additional target predict algorithms that incorporate thermodynamic stability have now also been developed. For example, the PITA algorithm [25] similarly calculated the thermodynamic stability of miR:mRNA interactions but additionally incorporated target site accessibility within mRNA 3′ UTRs (e.g., putative targets are severely penalized if a target site occurs within a portion of a mRNA predicted to be involved in a stable secondary structure). The next principle means of target prediction refinement was through requiring target site conservation between multiple species. To achieve this, most algorithms required that predicted target sites are located in homologous 3′ UTR regions. As an example, TargetScan [26]–[28] searched for conserved target sites in the alignments 28 vertebrate species 3′ UTRs. Next, due to several analyses suggesting that mRNAs are frequently targeted by individual miRs at multiple 3′ UTR positions, the PicTar algorithm [29]–[31] was designed to highly reward multiple binding sites within a 3′ UTR. In light of the vastly different strategies employed by these algorithms it is understandable why no individual method of target prediction has been widely accepted to date. Apparently each method carries both distinct advantages and limitations. For example, thermodynamic stability algorithms clearly rely on the accuracy of RNA structural modeling; as a result, conservation of target sites cannot identify species-specific target sites or binding sites for species-specific miRs. In addition, requiring multiple binding sites within individual 3′ UTRs discards a considerable number of false negatives.

Recently, strategies incorporating information beyond specific miR:target site interactions have begun to emerge. Because miRs likely regulate genes contributing to common biochemical pathways or biological processes, requiring functional relationships between putative targets has also been suggested as a means of target refinement. MirBridge [32] utilized this strategy by searching for disproportionately represented miR complementarities in functionally related genes and was primarily useful for identifying miRs regulating specific biological functions or pathways. Additionally, correlating miR and mRNA expression data makes a logical addition to target refinement as a pair of miR and mRNA have to be expressed in the same tissue in order to interact with each other. To achieve this, mimiRNA [33] correlated predicted human targets from TargetScan, miRanda, and PicTar with miR expression data.

### Related Work in Existing Bio-Ontologies

Bio-ontologies in Open Biomedical Ontologies (OBO) Foundry/Library: OBO Foundry/Library [34] encompasses numerous ontologies shared across various biological and biomedical domains. As of December 2013, there are ten formal bio-ontologies in the OBO Foundry and over 100 candidate bio-ontologies in the OBO Library, spanning topics from anatomy to ethology, and information about genes, their products and their corresponding phenotypes.

Bio-ontologies in National Center for Biomedical Ontology (NCBO) BioPortal: The goal of the NCBO is to support biomedical researchers through providing online tools and a Web portal (NCBO BioPortal [35]) to allow them to access, evaluate, and integrate distinct ontological resources across all areas of biomedical research and clinical practice. A major focus of the NCBO is to incorporate the use of bio-ontologies to facilitate the analysis of data generated from complex experiments.

Gene Ontology (GO): One of the most successful examples of applying ontological and semantic technologies into biomedical and biological research is GO Consortium [8], [9]. GO provides defined vocabularies for annotating the biological function, cellular role, and location of gene expression products in a highly structured way and in order to unify gene function nomenclature across species. Contributing members of GO Consortium each independently associate GO terms with biological molecules in the organism(s) they annotate. GO consists of three sub-ontologies (biological process, cellular component, and molecular function), and has significantly facilitated knowledge acquisition in protein structure and function studies.

Sequence Ontology (SO): SO [10], [11] is a defined vocabulary for the parts of a genomic annotation. SO provides a uniform, common set of terms and definitions for the parts of genome, as well as relationships obtained between those parts. This facilitates the exchange, analysis, and management of genomic data. Since SO strictly defines part-whole relationships, data interpreted by SO has become a standard substrate for automated reasoning, and SO sequence feature descriptions are compatible with extensional mereology operators.

PRotein Ontology (PRO): PRO [12] facilitates protein annotation helping guide new experiments. PRO components have been defined from evolutionary relationship-based protein classifications and deal with multiple proteins arising from a common gene (e.g., alternative splicing variants, proteins undergoing alternative from of cleavage, as well as other posttranslational modifications). PRO is a standard protein OBO Foundry ontology, with a core set of well-defined relationships facilitating semantic integration and machine reasoning compatibility.

OBO Relation Ontology (RO): OBO RO [36] is a set of relationships for standardization across multiple OBO Foundry ontologies and wider OBO Library ontologies. OBO RO incorporates central, upper-level, and domain-independent relationships such as *part_of* as well as relationships specific to biological systems such as *develops_from*.

Note that there is an ontology named “miRNAO” in NCBO BioPortal and OBO Library. Unfortunately, we have not found formal publications produced by the miRNAO group, and there are no documentations provided in the project website (https://code.google.com/p/mirna-ontology/). Therefore, we do not include a discussion of miRNAO in this paper.

### Related Work in (Semi)Automated Ontology Development

Translation-based algorithms translate or convert knowledge in certain formats, extensible markup language (XML) or unified modeling language (UML) for example, into ontologies. Gasevic et al. [37] introduced an approach based on extensible stylesheet language transformation (XSLT) to automatically generate an OWL model from an UML model. The architecture consisted of an ontology definition metamodel defined using meta object facility and the related ontology UML profile. An ontology can then be transformed from its XML metadata interchange format into an OWL description. The authors in [38] presented a methodology for generating ontologies out of existing XML data with relational origins. This methodology was implemented within an extensible XSLT framework, which can be used with arbitrary XSLT processors. OntoWiki [39] was designed as a tool providing support for agile, distributed knowledge engineering scenarios, an alternative user interface for the schema editor integrated in Powl. OntoWiki was implemented in the scripting language PHP, allowing to be easily deployed on most Web hosting environments. The ultimate goal of OntoWiki was to decrease the entrance barrier for projects and domain experts during their collaboration using semantic technologies. Pivk [40] proposed a framework, based on Hurst's table model, for automatic transformation of arbitrary table-like structures into ontological models.

Mining-based algorithms use natural language processing to mine text to obtain ontological entities and relationships. TERMINAE [41] aimed to assist in building an ontology, both from scratch and from text, without control by specific tasks. It integrated a terminological approach and an ontology management, defined concept types that reflected modeling choices, and contained traceability facilities. Nobécourt presented in [42] a method for acquiring knowledge from text. By using the 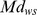 modeling language, links between words and conceptual primitives in the knowledge model can be kept, thus making it easier to maintain the knowledge model and its formalization Khan et al. [43] introduced an index structure to handle the challenge in keyword-based search (many documents convey desired semantic information without containing related keywords). Their methodology was based on the existing self-organizing tree algorithm (SOTA) that constructed a hierarchy from top to bottom. An ontology-learning approach was presented in [44], where WordNet lexicon resources were used to build standard OWL ontology models. The work by Lonsdale et al. [45] explored the common ground between the standardization of lexical/terminological resources and the use of conceptual ontologies for information extraction and data integration. Specifically, it aimed to improve the generation of extraction ontologies through the use of a comprehensive terminology database that has been represented in a standardized format. Balakrishna et al. presented a generalized procedure [46] to automatically extract semantic information from text resources. Semantically-rich domain ontologies can be created while keeping the manual intervention to a minimum. The Dresden Ontology Generator for Directed Acyclic Graphs (DOG4DAG) was introduced in [47], a system that supported the creation and extension of OBO ontologies by semi-automatically generating terms, definitions, and parent-child relations from text in PubMed, the Web, and PDF repositories. DOG4DAG was later integrated into OBO-Edit.

External knowledge-based algorithms build or enrich ontologies by using external resources (WordNet [78] for example). In the work by Moldovan et al. [48], different seed concepts selected from the financial domain were chosen, the relationship between concepts was then found using WordNet. Additionally, new concepts were integrated with an existing ontology. Finally the user can accept or decline concepts, patterns, and relationships. Agirre et al. [49] explored the possibility to exploit text on the Web to enrich concepts from existing ontologies. Documents related to certain concepts were retrieved from the Web, topic signatures for each concept in WordNet were then constructed, followed by building hierarchical clusters of these concepts. A generic method was presented in [50] for discovering a domain-tailored ontology from given intranet resources. The method was based on a given core ontology that was later extended with domain-specific concepts. The resultant ontology was pruned and restricted to certain applications using a corpus-based mechanism. Kong et al. [51] designed an automatic ontology-building system based on WordNet. The authors aimed to facilitate the ontology construction in a more consistent and easier manner. A method was introduced in [52] for ontology merging using WordNet. Two different approaches were presented. The horizontal approach was to analyze ontology mappings through similar concepts at the same level, and the vertical approach created rules from similar concepts at different levels.

### OMIT Naming Conventions

First, for imported concepts, properties, and relationships, we have kept their IDs (together with namespace prefixes, if any) and names unchanged even if we extended or customized them later on. For example, “bfo:Entity (*entity*),” “GO:0008150 (*biological_process*),” “SO:00000001 (*region*),” “PR:000000001 (*protein*),” “OBO_REL:part_of (*part_of*),” and so forth. The purpose was to further increase the interoperability across different ontologies, especially when it is necessary to cross-reference original sources. To handle the situation where original entities get updated in their home ontologies, we have followed the “minimum information to reference an external ontology term” (MIREOT) principle [82], [83] and utilized OntoFox [84], [85], a Web-based tool that fetches ontology terms and axioms, to facilite the ontology reuse.

For those concepts, properties, and relationships created by ourselves, the following naming conventions have been adopted.

- The prefix “OMIT:” was used for all concepts, properties, and relationships.
- Whenever possible, terms commonly adopted in cell biology (miR in particular) community were used for constructing meaningful names.
- For the purpose of being more computer-friendly, no spaces, points, periods, slashes, and brackets were allowed in names.
- Synonyms were utilized to keep track of variant biological terms that have the same semantics (intended biological meanings). There was no limit regarding how many synonyms one term can have, and synonyms did not have to follow the abovementioned naming conventions. Incorporating synonyms will facilitate the term search in the ontology.

### Human-Readable Definitions in OMIT

We have supplied human-readable definitions in OMIT for concepts, properties, and relationships. These definitions concisely and clearly state respective semantics. For example:

- The concept “OMIT:0000121 (*TumorMetastasis*)” — “Metastatic disease, which is the spread of a cancer from one organ or part to another non-adjacent organ or part.”
- The concept “OMIT:0000126 (*GeneticDisease*)” — “A general term for any disorder caused by a genetic mechanism, comprising chromosome aberrations (or anomalies), mendelian (or monogenic or single-gene) disorders, and multifactorial disorders.”
- The relationship “OMIT:hasValidation (*hasValidation*)” — “A target gene (either computationally predicted or biologically validated) may have one or more wet-lab experimental validations.”

Definitions were drawn from standard authoritative sources like Lewin's Genes XI [86] and The Molecular Biology of the Cell [87]. Human-readable definitions will further assist future communication across different research groups and therefore minimize potential confusion and disagreements regarding what a term is actually describing.

### Learning Problem and Weight-Learning Algorithm

#### Formal description of the learning problem

The learning problem described in Subsection “Ontology/Schema Alignment Algorithm” is formally defined as follows.

- Task *T*: Discover equivalent concept pairs between two ontologies/schemas.
- Performance measure *P*: Precision, Recall, F-Measure, and Overall measures regarding a manual alignment.
- Training experience *E*: A set of equivalent concept pairs provided by biologists.
- Target function *V*: A pair of concepts 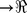
_._
- Target function representation: 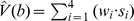
_._

#### ANN design and weight-learning algorithm

**Figure 7:**
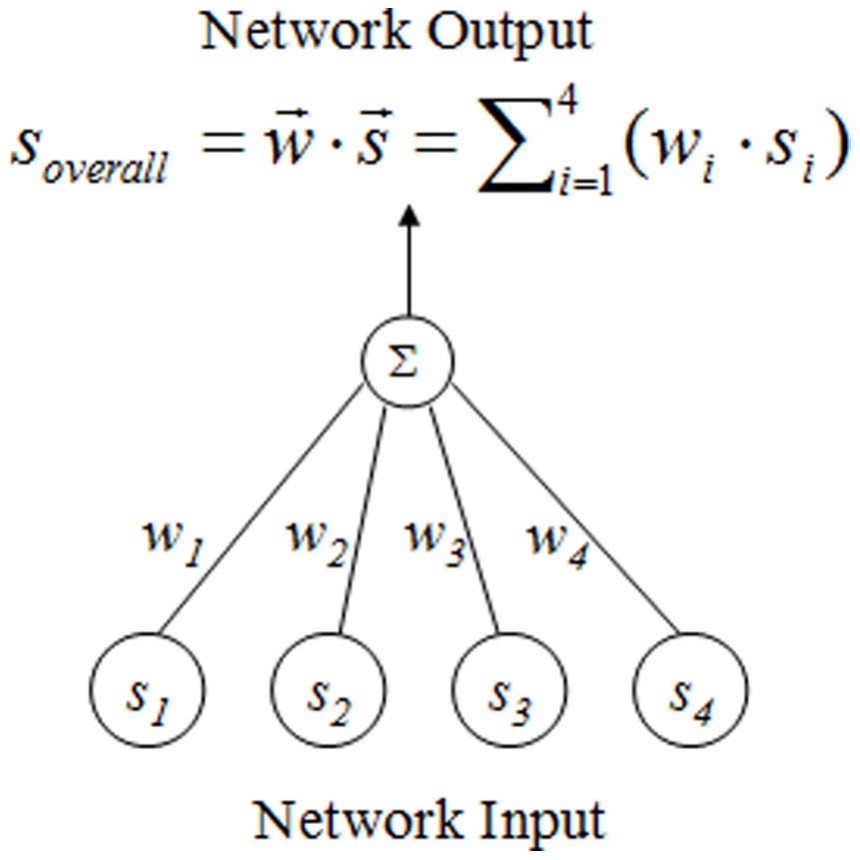
A two-layer, 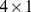 ANN designed for the learning problem.

**Figure 8:**
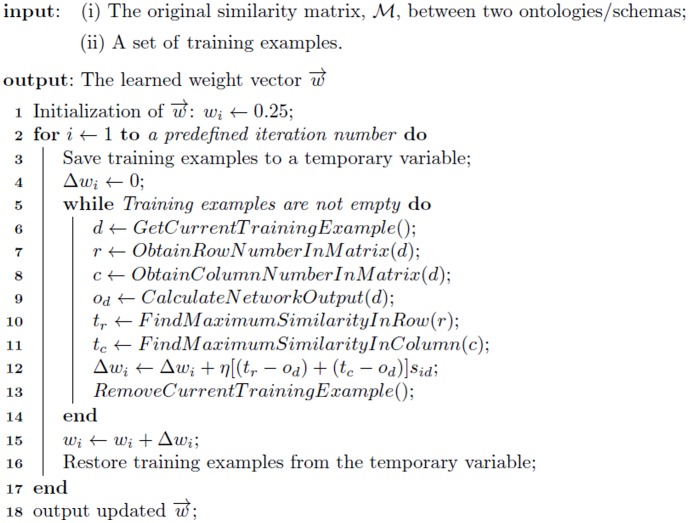
Pseudocode 1 — ANN Weight Learning.

A two-layer, 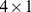 ANN (Figure 7) is designed for this learning problem. The hypothesis space is a four-dimensional space consisting of various weights (i.e., a collection of weight vectors). Gradient descent (delta rule) [88] is adopted as the training rule to find the weight vector (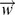) that best fits training examples, and the search strategy within the hypothesis space is to find 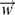 that is able to minimize the training error, *E*, regarding all training examples. According to current literature ([88] for example), a standard definition of 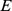 in a hypothesis is calculated as 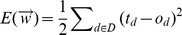, where 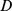 is the set of training examples, 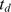 is the target output for a specific training example, 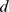, and 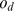 is the network output for this training example. A standard definition of the weight update rule is 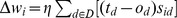, where 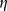 is the learning rate and 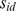 is the 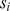 value for 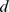. In this paper, the standard definition of *E* is customized as 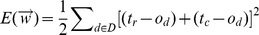, with 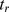 and 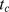 being maximum values for row 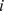 and column 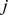 for a given cell 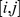 in the similarity matrix, respectively. The intuition here is that, a given pair of manually aligned concepts corresponds to a cell 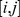 in the matrix; therefore, the value of cell 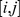 should be the maximum one in both row 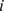 and column 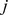. Accordingly, the weight update rule is re-designed as 
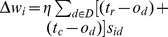
. The pseudocode for the proposed ANN learning is shown in Figure 8.

#### Complexity analysis

The time complexity of the ANN weight learning algorithm is analyzed as follows.

- First, the number of total iterations for both outer and inner loops is the multiplication of the iteration number (Line 2 in Figure 8) and the number of training examples (Line 5 in Figure 8), both of which are independent of the ontology/schema size.
- Second, the most time-consuming operations are Lines 10 and 11 in Figure 8, and both of which have a complexity of 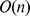, where 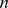 is the total number of concepts in the ontologies/schemas to be aligned. The time complexity of all other lines is 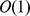. Therefore, the weight learning is scalable.

### Agglomerative Clustering Algorithm

#### The clustering algorithm

**Figure 9:**
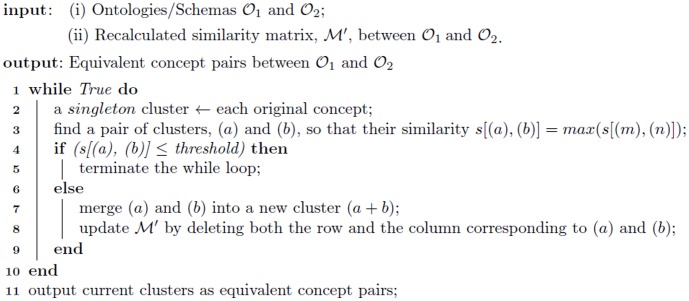
Pseudocode 2 — Agglomerative Clustering.

Once the updated 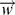 is obtained from the ANN, the similarity matrix is recalculated with learned, optimal weights. An agglomerative clustering algorithm is utilized to generate equivalent concept pairs. Initially, each concept is regarded as a *singleton* cluster, and clusters of two equivalent concepts can be merged with each other and form a new cluster. New clusters continue to be generated until the maximum similarity between any two clusters is below a predefined threshold. Finally, newly generated clusters are output as the set of equivalent concept pairs. The corresponding pseudocode is shown in Figure 9.

#### Complexity analysis

The time complexity of the agglomerative clustering algorithm is analyzed as follows.

- First, the most time-consuming operation is Line 3 in Figure 9, which has a complexity of 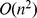, where 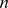 is the total number of concepts in the ontologies/schemas to be aligned. The complexity of all other operations is 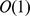.
- Second, the number of total iterations for the while loop (Line 1 in Figure 9) is determined by the number of equivalent concept pairs between two input ontologies/schemas. Even if two ontologies/schemas have a very large fraction of overlapping concepts, the worst-case scenario is that, the number of total iterations will be 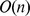, so the total time complexity is still a polynomial of the total number of concepts.

## Conclusions

Significant barriers exist during biologists' conventional miR knowledge discovery because large amounts of data sources need to be explored and these data sources are semantically heterogeneous among each other. The situation has been further worsened by the fact that naming conventions for miR data are still in their infancy and not yet uniformly adopted. Emerging semantic technologies, which are based upon domain ontologies, are proved to be able to render critical assistance to this problem. Our previous research has investigated the construction of OMIT, the very first of its kind that formally encodes miR domain knowledge. Although it is essential to have a manual component contributed by domain experts when building ontologies, relying on a purely manual development has many challenges. According to these insights, we proposed in this paper a semi-automated ontology development methodology, which makes use of machine intelligence, considers miR domain-dependent and domain-independent properties/relationships, is scalable, and has significantly reduced human efforts. Experiments have been conducted to thoroughly evaluate our methodology. Our contributions can be summarized as: (i) We have continued the development and critical improvement of OMIT, solidly based on our previous research outcomes. (ii) We have explored effective and efficient algorithms with which the ontology development can be seamlessly combined with machine intelligence and be accomplished in a semi-automated manner, thus significantly reducing large amounts of human efforts. Note that to semantically annotate miR-related data is by itself an important research issue but is beyond the scope of this paper.

We plan to continue the development and refinement of OMIT. An example future work is to consider more miR domain-dependent relationships to further improve the effectiveness of the alignment algorithm. Another example is to include other related bio-ontologies during the alignment process to further enrich the resultant ontology. Currently, GO, SO, and PRO teams are collaborating on the OMIT project. We will involve an even wider range of experimental biologists and bioinformaticians in the future. As a result, relevant research communities can make respective, collaborative contributions to OMIT.
